# Unsupervised Cerebrovascular Segmentation of TOF-MRA Images Based on Deep Neural Network and Hidden Markov Random Field Model

**DOI:** 10.3389/fninf.2019.00077

**Published:** 2020-01-10

**Authors:** Shengyu Fan, Yueyan Bian, Hao Chen, Yan Kang, Qi Yang, Tao Tan

**Affiliations:** ^1^School of Sino-Dutch Biomedical and Information Engineering, Northeastern University, Shenyang, China; ^2^Neusoft Research of Intelligent Healthcare Technology, Co. Ltd., Shenyang, China; ^3^Engineering Research Center for Medical Imaging and Intelligent Analysis, National Education Ministry, Shenyang, China; ^4^Department of Biomechanical Engineering, University of Twente, Twente, Netherlands; ^5^Department of Radiology, Xuanwu Hospital, Capital Medical University, Beijing, China; ^6^Department of Biomedical Engineering, Eindhoven University of Technology, Eindhoven, Netherlands

**Keywords:** deep neural network, hidden Markov random field model, cerebrovascular segmentation, magnetic resonance angiography, unsupervised learning

## Abstract

Automated cerebrovascular segmentation of time-of-flight magnetic resonance angiography (TOF-MRA) images is an important technique, which can be used to diagnose abnormalities in the cerebrovascular system, such as vascular stenosis and malformation. Automated cerebrovascular segmentation can direct show the shape, direction and distribution of blood vessels. Although deep neural network (DNN)-based cerebrovascular segmentation methods have shown to yield outstanding performance, they are limited by their dependence on huge training dataset. In this paper, we propose an unsupervised cerebrovascular segmentation method of TOF-MRA images based on DNN and hidden Markov random field (HMRF) model. Our DNN-based cerebrovascular segmentation model is trained by the labeling of HMRF rather than manual annotations. The proposed method was trained and tested using 100 TOF-MRA images. The results were evaluated using the dice similarity coefficient (DSC), which reached a value of 0.79. The trained model achieved better performance than that of the traditional HMRF-based cerebrovascular segmentation method in binary pixel-classification. This paper combines the advantages of both DNN and HMRF to train the model with a not so large amount of the annotations in deep learning, which leads to a more effective cerebrovascular segmentation method.

## Introduction

According to the World Health Organization (WHO) report on the global burden of stroke, adult stroke mortality rate has reached 39% (Kim and Johnston, 2011). The pathogenesis of stroke is commonly associated to disorders in human cerebrovascular system (Arvanitakis et al., 2016), and hence an accurate cerebrovascular segmentation is of vital importance for further diagnosis and also for computer-aided diagnosis (CAD) (Yan and Kassim, 2005). Time-of-Flight magnetic resonance angiography (TOF-MRA) is the most widely used imaging technique to observe a complete cerebrovascular tree, because no contrast agent is required for this technique. Automated and accurate cerebrovascular segmentation from TOF-MRA images is beneficial to quantitatively analyze cerebrovascular disorders, such as the estimation of vascular stenosis rate, and also to assess cerebral collateral circulation (Lee et al., 2005; Bicakci et al., 2006).

In the past few years, many methods for extracting cerebrovascular trees were developed based on deformable models (Kavsak et al., 2000; Aylward and Bullitt, 2002; Yim et al., 2003; Yan and Kassim, 2006; Lorigo et al., 2010), statistical models (Wilson and Noble, 1997; Zhang et al., 2001; Gan et al., 2004; Elbaz et al., 2005; Hassouna et al., 2006), and deep neural network (DNN) (Wilson and Noble, 1997; Zhang et al., 2001; Gan et al., 2004; Elbaz et al., 2005; Hassouna et al., 2006). From deformable model-based methods, geodesic active contours is a typical representative method, which fits topological structures of blood vessels in TOF-MRA images with level-set techniques (Lorigo et al., 2010). Yan et al. proposed an effective segmentation method using capillary active contours, which extended geodesic active contours to capillaries modeled on the physical phenomenon of capillary actions (Yan and Kassim, 2006). However, deformable models can easily have leakage around the edge (Angelini et al., 2005; Cengizler et al., 2014). The leakage gets into the area outside of blood vessels during iterative optimization, especially at the end and the stenotic parts of blood vessels. Moreover, in our opinion these models may have a poor performance on TOF-MRA images with inhomogeneity. Statistical model-based methods extract cerebrovascular trees by fitting intensity distributions of different tissues into statistical models such as Gaussian mixture models. Hidden Markov Random Field (HMRF) and Expectation-Maximization (EM) framework were also widely used to segment blood vessels and brain tissue from MR images (Wilson and Noble, 1997; Zhang et al., 2001; Gan et al., 2004; Elbaz et al., 2005; Hassouna et al., 2006). Zhang et al. (2001) firstly introduced HMRF model and EM algorithms to segment gray matter (GM), white matter (WM) and cerebrospinal fluid (CSF) in brain MR images. Hassouna et al. (2006) proposed a 3D cerebrovascular segmentation method using stochastic models, which described the intensity histogram of MRA images by a finite mixture model consisting of one Rayleigh and two normal distributions. These stochastics models also estimated spatial contextual information using 3D HMRF, then they segmented blood vessels by optimizing HMRF and EM framework (Hassouna et al., 2006). A drawback of the abovementioned statistical model-based methods is that their segmentation performances significantly depend on the adaptation between statistical model and intensity histogram of MR images, and therefore their performances are sensitive to the intensity distortion of TOF-MRA images.

Deep neural network-based cerebrovascular segmentation methods have been proposed with great successes in semantic segmentation (Chen et al., 2017; Nakao et al., 2017; Phellan et al., 2017; Sahin and Ünal, 2017). Chen et al. (2017) proposed a convolutional auto-encoder named Y-net to segment intracranial artery in MRA images, of which dice similarity coefficient (DSC) reached a value of 0.828. Phellan et al. (2017) built a DNN model consisting of two convolutional layers and two fully connected layers to extract cerebrovascular trees, of which the achieved DSC ranged from 0.764 to 0.786. These DNN-based vessel segmentation methods have outperformed the abovementioned traditional machine learning methods, but the training sets of DNN-based methods mostly consisted of TOF-MRA images from only one type of MR scanner with the same resolution. According to prior experiences (Simonyan and Zisserman, 2014; Badrinarayanan et al., 2015; Ronneberger et al., 2015), DNNs need to be trained with a large amount of various TOF-MRA images annotated manually in order to keep a good performance for TOF-MRA images with different resolutions from different devices. However, since human cerebrovascular system is complicated and huge, a large amount of manual annotations of TOF-MRA images is very expensive to obtain.

Given all the aforementioned limitations of existing cerebrovascular segmentation algorithms, we propose a new unsupervised cerebrovascular segmentation framework which combines DNN with HMRF model. It does not require a large amount of manual annotations and achieves great performance for TOF-MRA images for different devices and with different resolutions. We compared two frameworks: HMRF + SegNet2D and HMRF + U-Net3D. These two frameworks are assessed on TOF-MRA images with different resolutions from different devices. The remaining parts of this paper are organized as follows. Section “Materials and Methods” provides the mathematical details of the HMRF and EM algorithm, and the architectures of SegNet and U-Net. In section “Experiments,” the experimental dataset and experimental setting are described, while section “Results” shows the various experiments performed to evaluate the performance of the proposed method. This is followed by a discussion about our approach in section “Discussion.” Finally, we give a conclusion in section “Conclusion.”

## Materials and Methods

### Unsupervised HMRF + DNN-Based Cerebrovascular Segmentation

In previous studies, DNN-based cerebrovascular segmentation methods have significantly outperformed traditional methods (Simonyan and Zisserman, 2014; Badrinarayanan et al., 2015; Ronneberger et al., 2015). Since the human cerebrovascular system has the intricate shape and high inter-individual difference, manual annotations of cerebrovascular trees take too much time. Thus, researchers often use a small amount of TOF-MRA images to evaluate the performance of DNN-based methods even though they necessitate great amount of data. To solve this problem, we propose an unsupervised cerebrovascular segmentation framework by adding a HMRF-based pre-segmentation method before DNN architectures.

**FIGURE 1:**
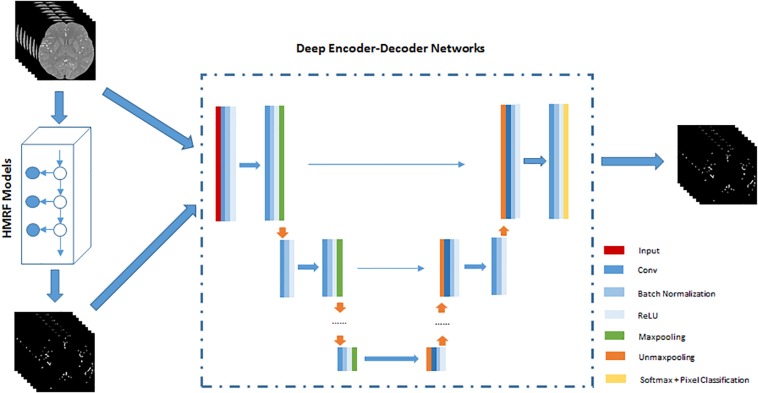
An illustration of the workflow of unsupervised HMRF + DNN-based cerebrovascular segmentation method. Preprocessed TOF-MRA images are pre-segmented to extract roughly cerebrovascular system using HMRF method, and then these images and rough masks of blood vessels are used to train deep encoder-decoder network. Finally, blood vessels are pixel-classified by deep encoder-decoder network.

The HMRF + DNN framework for cerebrovascular segmentation mainly consists of two parts, pre-segmentation of blood vessels using HMRF and DNN architecture. In the pre-segmentation part, we use HMRF technique to extract brain blood vessels based on their intensity and spatial information in TOF-MRA images. Generally, the brain blood vessels extracted using HMRF method are thick artery blood vessels due to the fact that thick blood vessels have strong intensity differences from brain tissue. Although cerebrovascular system has the intricate shape, the difference between thick and small blood vessels is mainly found in the radius of blood vessels or spatial scale. Therefore, the HMRF-based segmentation result includes most of the features of blood vessels except spatial-scaling feature, while the spatial-scaling feature can be learnt by setting max-pooling layers in the DNN architecture. The second part, DNN architecture, is trained by the results of pre-segmentation of blood vessels. In this paper, 2D SegNet and 3D U-Net are adopted to perform cerebrovascular segmentation. The workflow of the unsupervised HMRF + DNN-based cerebrovascular segmentation method is illustrated in Figure 1.

### Cerebrovascular Segmentation Method Based on HMRF Model and EM Algorithm

Hidden Markov random field model is extended by Markov random filed (MRF) and hidden Markov model (HMM) (Zhang et al., 2001), which consists of a sequence of statistical states hidden in MRF but observable in the observation field. In TOF-MRA images, the spatial information can be described by the associativity between the neighboring pixels, while the intensity information can be represented into Gaussian mixture models in each region-of-interest [e.g., brain tissue, vascular trees, and CSF]. HMRF model can extract cerebrovascular trees using both the spatial and intensity information.

Let **S** = {1,2,3,…,*S*} represent the set of indices of voxels in TOF-MRA images, **X** = {*X*_*i*_,*i* ∈ *S*} and **Y** = {*Y*_*i*_,*i* ∈ *S*} represent the sets of label and image, **L** = {1,2,3,…,*L*} be the set of region classes in TOF-MRA images, where S is the number of voxels and L is the number of region classes. If we assume that **X** and **Y** are two random fields and any pair of (*X*_*i*_,*Y*_*i*_) is the pairwise independence, the joint probability distribution of (**Y**,**X**) is:

(1) $$\text{P}\,\left(Y,X\right)=\prod_{i\in S}P\,\left(Y_{i},X_{i}\right)$$

According to the MRF theory (Zhang et al., 2001), the labels in S are related to their neighborhood system, which is defined as *N* = {*N*_*i*_,*i* ∈ *S*}, where *N*_*i*_ is the set of labels neighboring *i*,*i*∉*N*_*i*_ and *i* ∈ *N*_*j*_≡*j* ∈ *N*_*i*_. A Markov random field X can be represented with a neighborhood system if and only if:

(2) P⁢(x)>0,∀x∈X

(3) $$\text{P}\,\left(x_{i}|x_{S-\left\{i\right\}}\right)=P\,\left(x_{i}|x_{N_{i}}\right)$$

where S−{*i*} is the set of indices of voxels except {*i*} in TOF-MRA images. Thus the above joint probability (1) can be reformed into the following expression:

$$\text{P}\,\left(Y,X\right)=\prod_{i\in S}P\,\left(Y_{i},X_{i}|X_{N_{i}}\right)$$

(4) $$=\prod_{i\in S}P\,\left(Y_{i}|X_{i}\right)\,P\,\left(X_{i}|X_{N_{i}}\right)$$

and the marginal probability distribution of *Y*_*i*_ is:

$$\text{P}\,\left(Y_{i}|X_{N_{i}},\theta \right)=\sum_{l\in L}P\,\left(Y_{i},l|X_{N_{i}},\theta \right)$$

(5) $$=\sum_{l\in L}P\,\left(Y_{i}|l,X_{N_{i}},\theta_{l}\right)\,P\,\left(l|X_{N_{i}}\right)$$

Where θ_*l*_ = (μ_*l*_,σ_*l*_)^*T*^, respectively, μ_*l*_ and σ_*l*_ represent the expectation and variance of Gaussian distribution.

According to the histogram of intensities of TOF-MRA images, we assume that the conditional probability distribution of each region class is a Gaussian distribution. Given *Y*_*i*_ = *l*, *X*_*i*_ follows a conditional probability distribution:

(6) $$\text{P}\,\left(Y_{i}|l\right)=g\,\left(Y_{i};\theta_{l}\right),\forall l\in L$$

(7) $$g\,\left(Y_{i};\theta_{l}\right)=\frac{1}{\sqrt{2\,\pi \,\sigma_{l}^{2}}}\,\exp\left(-\frac{{\left(Y_{i}-\mu_{l}\right)}^{2}}{2\,\sigma_{l}^{2}}\right)$$

Thus, the Gaussian HMRF model is represented as:

(8) $$\text{P}\,\left(Y_{i}|X_{N_{i}},\theta \right)=\sum_{l\in L}g\,\left(Y_{i};\theta_{l}\right)\,P\,\left(l|X_{N_{i}}\right)$$

To find a labeling $\hat{X}$ of TOF-MRA images, it can be used to estimate the ground truth labeling *X*^∗^ using the maximizing a posterior (MAP) criterion:

(9) $$\hat{X}=\arg\max_{x\in X}\left\{P\,\left(y|x\right)\,P\,\left(x\right)\right\}$$

The prior probability of each voxel is different. According to the Hammersley system theorem (Hammersley and Clifford, unpublished), since **X** is considered as a MRF, its prior probability can be formulated as:

(10) $$\text{P}\,\left(X\right)=Z^{-1}\,\exp\left(-U\,\left(X\right)\right)$$

where Z is the partition function which is a normalizing constant, and U(**Y**) is an energy function:

(11) $$\text{U}\,\left(X\right)=\sum_{c\in C}V_{c}\,\left(X\right)$$

where *V*_*c*_(**X**) is the clique potential function.

(12) $$\text{P}\,\left(\text{Y}|\text{X}\right)=\frac{1}{Z^{\prime }}\,\exp\left(-U\,\left(\text{Y}|\text{X}\right)\right)$$

where *U*(**Y**|**X**) is the likelihood energy.

$$U\,\left(\text{Y}|\text{X}\right)=\sum_{i\in S}U\,\left(Y_{i}|X_{i}\right)$$

(13) $$=\sum_{i\in S}\left[\frac{{\left(Y_{i}-\mu_{X_{i}}\right)}^{2}}{2\,\sigma_{X_{i}}^{2}}+\log\left(\sigma_{X_{i}}\right)\right]$$

and *Z*′ = (2π)^(*N*/2)^. Thus, it has an obvious relationship *log*⁡(*P*(**X**|**Y**))∝−*U*(**X**|**Y**)where:

(14) U⁢(X|Y)=U⁢(Y|X)+U⁢(X)+c⁢o⁢n⁢s⁢t

is called the posterior energy. Thus, the labeling $\hat{X}$ can be estimated by minimizing the posterior energy function:

(15) $$\hat{X}=\arg\min_{x\in X}\left\{U\,\left(\text{Y}|\text{X}\right)+U\,\left(X\right)\right\}$$

According to the above derivation, the problem of the optimal segmentation is equivalent to minimizing the posterior energy function. To solve the equation (9), we estimate the optimal parameters of HMRF model using EM algorithm, which is an iterative optimal algorithm to solve the problem of the estimation of maximum likelihood or posterior. For more details on EM algorithm, kindly refer to (Dempster et al., 1977). The brief description of EM algorithm for optimizing HMRF model is given as follows.

Start Initialize the estimated parameters θ^0^.

E-step Calculate the expectation of log joint probability:

$$\text{Q}\,\left(\theta |\theta^{\left(t\right)}\right)=\varepsilon \,\left[\log\left(P\,\left(X,Y|\theta \right)\right)|Y,\theta^{\left(t\right)}\right]$$

$$=\sum_{x\in X}P\,\left(x|y,\theta^{\left(t\right)}\right).\log P\,\left(x,y|\theta \right)$$

M-step Maximize the log joint probability to estimate the new parameters θ^(t+1)^:

$$\theta^{\left(t+1\right)}=\arg\max_{\theta }\text{Q}\,\left(\theta |\theta^{\left(t\right)}\right)$$

$$\mu_{l}^{\left(t+1\right)}=\frac{\sum_{i\in S}P^{\left(t\right)}\,\left(l|Y_{i}\right)\,Y_{i}}{\sum_{i\in S}P^{\left(t\right)}\,\left(l|Y_{i}\right)}$$

$${\left(\sigma_{l}^{\left(t+1\right)}\right)}^{2}=\frac{\sum_{i\in S}P^{\left(t\right)}\,\left(l|Y_{i}\right)\,{\left(Y_{i}-\mu_{l}\right)}^{2}}{\sum_{i\in S}P^{\left(t\right)}\,\left(l|Y_{i}\right)}$$

where $P^{\left(t\right)}\,\left(l|Y_{i}\right)$ is estimated by the equation (7) in MRF-MAP estimation procedure.

Update assign θ^(*t* + 1)^ to θ^(t)^ and repeat from E-step.

### Deep Convolutional Encoder-Decoder Network

**FIGURE 2:**
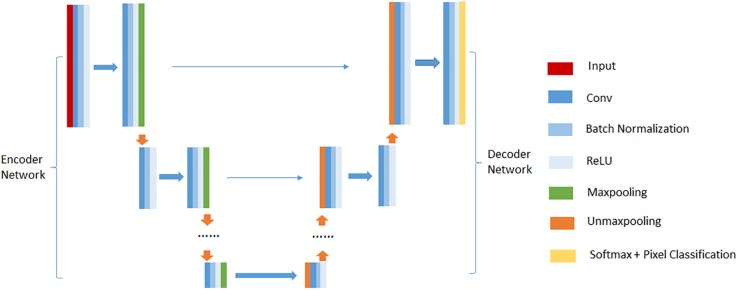
An illustration of the common DCEDN architecture.

Deep convolutional encoder-decoder network (DCEDN) is a new deep convolutional neural network resulted from modifying the fully convolutional network (FCN) (Long et al., 2015). It can provide more precise segmentation results with few training datasets. The well-known architectures of DCEDN include SegNet (Badrinarayanan et al., 2015), U-Net (Ronneberger et al., 2015), and their main ideas consist of trying to map low resolution features to input resolution for pixel-wise classification. Their common architecture is illustrated in Figure 2. There is no fully connected layers in their architectures. They mainly contain two parts, encoder network and their corresponding decoder network. Encoder network is designed to extract feature maps of input images, while decoder network up-samples low resolution feature maps into the input resolution. The encoder network consists of a few convolutional layers, batch normalization layers, rectified linear unit (ReLU) layers and max-pooling layers. In common, the encoder network is designed as the traditional architecture for object classification [e.g., VGG16 (Simonyan and Zisserman, 2014)], while the difference compared to traditional network for object classification is to memorize the max-pooling indices in SegNet or the feature maps in U-Net. Decoder network usually contains the same number of up-sampling layers, convolutional layers, batch normalization layers and ReLU layers as the encoder network. Up-sampling layers is used to up-sample the feature maps from the encoder network. Finally, the feature maps with the input resolution are pixel-classified by a soft-max layer, and the probabilities for each class are output.

**FIGURE 3:**
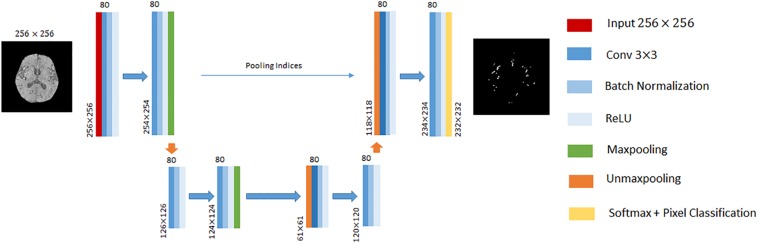
An illustration of SegNet architecture adopted in this paper. The different layers is indicated by boxes with different colors. The input of this architecture is 256×256 2D TOF-MRA image. The input size of each convolutional block is denoted at the left bottom, and the number of channels on the top.

In this paper, we performed cerebrovascular segmentation from TOF-MRA images based on 2D SegNet (Badrinarayanan et al., 2015) and 3D U-Net (Çiçek et al., 2016). The DCEDN and SegNet architecture adopted in this paper is illustrated in Figures 2, 3. The input of SegNet architecture consists of each 2D slice of TOF-MRA images which is resized into 256×256. The main structure of the 2D SegNet consists of 8 convolutional layers, 8 batch normalization layers, 8 ReLU layers, 2 max-pooling layers, and 2 up-sampling layers and a soft-max layers. Each convolutional layer contains 80 filters with 3×3 voxels receptive field in a 1 voxel stride sliding. The batch normalization layer after each convolutional layer helps improve the convergence speed of SegNet, and the ReLU layer can reduce the impact of the backpropagation vanishing problem. Finally, the pixel classification is processed in the soft-max layer.

**FIGURE 4:**
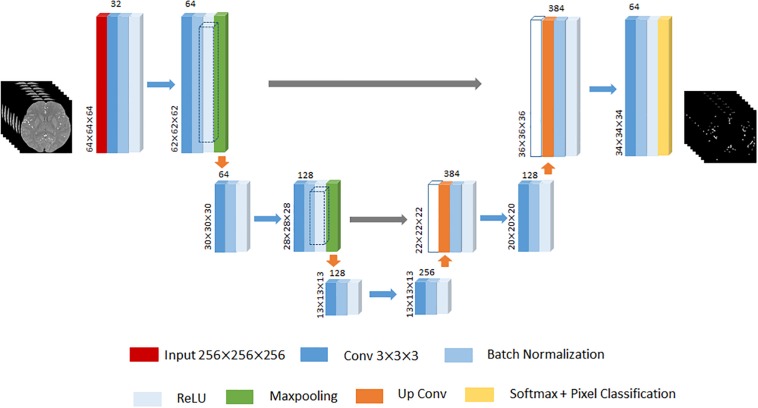
An illustration of 3D U-Net architecture. The different layers is indicated by boxes with different colors. The input of this architecture is 64×64×64 3D TOF-MRA volume region. The input size of each convolutional block put at the left bottom, and the number of channels put on the top. The gray arrow represents the corresponding feature concatenation.

Given that 3D U-Net has achieved remarkable successes in various biomedical segmentation tasks (Ronneberger et al., 2015; Çiçek et al., 2016; Tong et al., 2017), we chose 3D U-Net as our framework example to evaluate the performance of the proposed method. One of differences with 2D SegNet architecture is that the input is the 3D volume region of TOF-MRA images, while each TOF-MRA images is patched into 64×64×64 because of the limitation of the memory. The 3D U-Net architecture designed in this paper contains the encoder network to encode the valid feature and the decoder network to up-sample the low resolution feature back to the input resolution. The encoder network consists of 6 convolutional layers, and each of them is followed by a batch normalization layer and a ReLU layer, and 2 max-pooling layers to change the feature resolution. The decoder network consists of 2 up-convolutional layers and 2 convolutional layers, and each of them is followed by a batch normalization layer and a ReLU layer like the encoder work. The feature up-sampled by the up-convolutional layer is concatenated with the correspondingly cropped feature before the batch normalization layer. Finally, soft-max layer performs the voxel-based classification and outputs the probabilities for each cluster. An illustration of the 3D U-Net used in this paper is displayed in Figure 4.

### HMRF + DNN Framework Training

The training of the HMRF + DNN framework for cerebrovascular segmentation includes two parts, auto-labeling ROI of TOF-MRA images using the HMRF model and the training of the DNN model. According to the intensity distribution of TOF-MRA images, the intensity distributions of brain tissue and blood vessels can be approximately represented as Gaussian distributions. Then, we constructed two Gaussian HMRF models to automatically extract the blood vessels in the preprocessed TOF-MRF images. The intensity of the background of TOF-MRA images is zero through preprocessing, so we labeled the background into an individual class in order to improve the performance of the algorithms. Thus, in the first part of HMRF + DNN framework, we labeled each TOF-MRF images into three classes regions, background, brain tissue and blood vessels using HMRF model method.

The second part of the HMRF + DNN framework is the DNN training using TOF-MRA images and their labeling resulted from the first part. We constructed two architectures in this paper to segment cerebrovascular trees in TOF-MRA images, respectively, 2D SegNet and 3D U-Net. The input image of 2D SegNet consists of each 2D slice of TOF-MRA images, whereas in the 3D U-Net, the input consists of 3D volume region. To improve the performance of 2D SegNet in cerebrovascular segmentation, we built the HMRF + SegNet2D model with three sub-SegNets which were, respectively, trained by 2D TOF-MRA images in axial, sagittal and coronal directions, which is based on the neurophysiology theory that cerebrovascular systems in different individuals have similarly 3D topological structures. Then, the final probability map was estimated by averaging the probability maps from these three 2D SegNets. The loss function over the whole training datasets was minimized through a mini-batch gradient descent approach, and the minimum of batch size was 50 inputs. The learning process goes through 50 epochs with a learning rate of 0.001 and a gradient momentum of 0.9. In 3D U-Net learning process, there are the same parameter settings in epoch number, learning rate and gradient momentum, but the minimum of batch size is set as 8 because of the limitation of memory.

## Experiments

### Data Preparation and Image Pre-processing

In this study, we collected 100 TOF-MRA cases including 30 healthy cases and 70 stroke cases, which are used to train and evaluate the performance of different segmentation methods. 60 TOF-MRA cases among the total dataset were acquired on a 1.5T Discovery MR750 GE MRI scanner without contrast agent at a parameter setting of a TE = 2.6 ms, a TR = 22 ms and a flip angle = 20 degree. The voxel size of each 1.5T TOF-MRA is 0.43×0.43×0.59*mm*^3^, and each volume contains 512×512×164 voxels. The other 40 TOF-MRA cases were acquired on a 3T Verio SIEMENS MRI scanner without contrast agent at a parameter setting of a TE = 3.6 ms, a TR = 21 ms and a flip angle = 18 degree, and their voxel size is 0.30 mm × 0.30 mm × 0.7382 mm and each volume size is 616×768×136.

**FIGURE 5:**
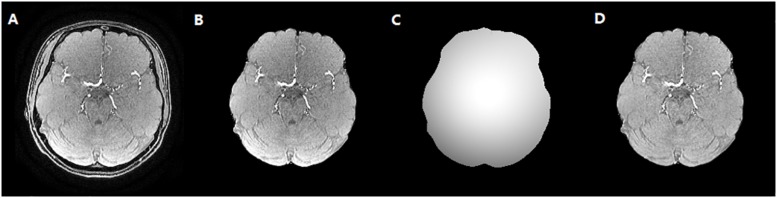
The skull stripping and bias correction results. **(A)** Original TOF-MRA image. **(B)** Skull stripping result. **(C)** Bias field. **(D)** Bias corrected TOF-MRA image.

To reduce the impact of the brain skull on the cerebrovascular segmentation, the dataset was preprocessed to remove brain skull using the BET2 method (Wels et al., 2009), which was followed by a bias correction using multiplicative intrinsic component optimization (MICO) algorithm (Li et al., 2014). Then, maximum intensity projection (MIP) images in axial, sagittal and coronal axis were acquired with a MIP algorithm. The vessels in MIP images of each case were manually segmented by medical experts to evaluate the performance of algorithm, which is illustrated in Figure 5.

### Hardware Settings

In this paper, our experiments were implemented, respectively, using MATLAB 2017b and Python 3.0 in Window 10 OS. Environments were made on a desktop computer with eight Intel(R) Xeon(R) CPU E5-1620 v4 @ 3.50 GHz processors, 32 GB of RAM memory and NVIDIA GeForce GTX 1080.

### Evaluation Method

Since manual annotations for 100 cases of TOF-MRA images need too much time, we manually segmented the vessels in MIP images of each case in axial, coronal and sagittal directions. We first adjusted the threshold, to segment high pixels, and then modified it manually, focusing on the edges and the ends of the vessels, as well as some small vessels. The performance of the proposed method in cerebrovascular segmentation is evaluated by comparing MIP post-processed binary images resulted from the proposed method with manual annotations, respectively, in axial, coronal and sagittal directions. Because MIP images in axial, coronal and sagittal directions contain the most information of blood vessels, the comparison of MIP binary images in axial, coronal and sagittal directions is able to indicate the difference of cerebrovascular segmentation between the proposed method and manual annotations. Therefore, the binary classification performance of the proposed method is evaluated by accuracy, sensitivity, specificity, precision, and DSC (Dice, 1945) which is defined as $\text{DSC}=\frac{2\,\left|A\cap B\right|}{\left(\left|A\right|+\left|B\right|\right)}$, where A and B is, respectively, the ground-truth and segmentations of DCEDN. DSC ranges from 0 to 1.

## Results

We evaluated the performance of HMRF + DNN framework in cerebrovascular segmentation to compare segmentation results using HMRF, HMRF + SegNet2D, and HMRF + U-Net3D methods. We separated all of the 100 TOF-MRA data into training and testing datasets. We randomly chose 20 TOF-MRA data from 1.5T GE scanner and 20 TOF-MRA data from 3.0T SIEMENS scanner to build up the training dataset, while the other 60 TOF-MRA data were assigned to the testing dataset. HMRF + SegNet2D and HMRF + U-Net3D were trained using the training dataset. Then, testing dataset was segmented by HMRF, trained HMRF + SegNet2D and trained HMRF + U-Net3D methods, and their results were evaluated according to the above mentioned method.

**FIGURE 6:**
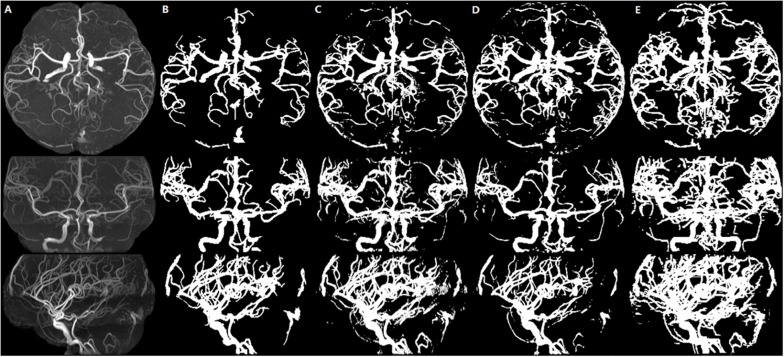
Healthy person. An illustration of axial MIP images of cerebrovascular segmentation results through HMRF, HMRF + SegNet2D, HMRF + U-Net3D and manual annotations. **(A)** TOF-MRA MIP images in axial, coronal and sagittal three axes. **(B)** MIP images of HMRF result in three axes. **(C)** MIP images of HMRF + SegNet2D result in three axes. **(D)** MIP images of HMRF + U-Net3D result in three axes. **(E)** Manual annotations in three axes.

**FIGURE 7:**
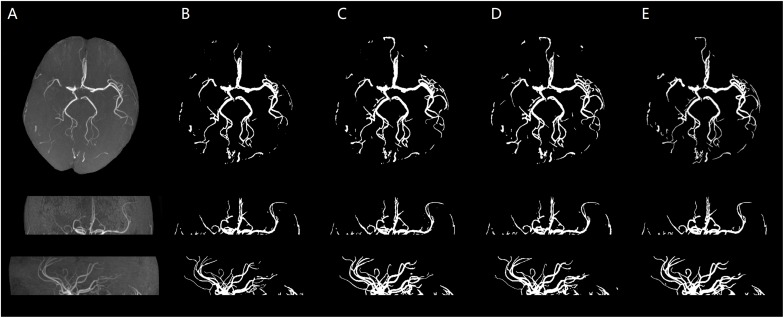
Stroke patient. An illustration of axial MIP images of cerebrovascular segmentation results through HMRF, HMRF + SegNet2D, HMRF + U-Net3D and manual annotations. **(A)** TOF-MRA MIP images in axial, coronal, and sagittal three axes. **(B)** MIP images of HMRF result in three axes. **(C)** MIP images of HMRF + SegNet2D result in three axes. **(D)** MIP images of HMRF + U-Net3D result in three axes. **(E)** Manual annotations in three axes.

We illustrate a case of healthy person of axial MIP images of segmentation results of HMRF, HMRF + SegNet2D and HMRF + U-Net3D in TOF-MRF images in Figure 6, and a case of stroke patient in Figure 7. The evaluation table for cerebrovascular segmentation results of HMRF, HMRF + SegNet2D and HMRF + U-Net3D in testing dataset are reported in Table 1. We also show the evaluation results of healthy people and stroke patients in Tables 2, 3, respectively. The DSC values were estimated by comparing the MIP images of cerebrovascular segmentation in axial, coronal and sagittal directions with the corresponding manual ground-truths. Values in each column were the average among the testing dataset.

**TABLE 1:** Evaluation of cerebrovascular segmentation evaluation in all samples.

**Methods**	**Accuracy**	**Sensitivity**	**Specificity**	**Precision**	**DSC**
HMRF	0.9947	0.5073	**0.9997**	**0.9472**	0.6141 ± 0.155
HMRF +	0.9982	**0.7967**	0.9991	0.7981	**0.7966** ± **0.035**
SegNet2D					
HMRF +	**0.9983**	0.7620	0.9993	0.8405	0.7941 ± 0.048
U-Net3D					

*The bold values mean the best performance by different method.*

**TABLE 2:** Evaluation of cerebrovascular segmentation evaluation in healthy people.

**Methods**	**Accuracy**	**Sensitivity**	**Specificity**	**Precision**	**DSC**
HMRF	0.9945	0.5072	**0.9996**	**0.9388**	0.6139 ± 0.157
HMRF +	0.9982	**0.7967**	0.9991	0.7981	**0.7952** ± **0.065**
SegNet2D					
HMRF +	**0.9983**	0.7620	0.9993	0.8405	0.7938 ± 0.058
U-Net3D					

*The bold values mean the best performance by different method.*

**TABLE 3:** Evaluation of cerebrovascular segmentation evaluation in stroke patients.

**Methods**	**Accuracy**	**Sensitivity**	**Specificity**	**Precision**	**DSC**
HMRF	0.9948	0.5124	**0.9997**	**0.9567**	0.6192 ± 0.146
HMRF +	0.9983	**0.8006**	0.9991	0.8028	**0.7969** ± **0.028**
SegNet2D					
HMRF +	**0.9984**	0.7708	0.9993	0.8441	0.7947 ± 0.041
U-Net3D					

*The bold values mean the best performance by different method.*

## Discussion

Deep neural network models are supervised deep learning models which have been widely used to perform object recognition and segmentation. They are commonly trained with a large amount of images labeled by humans. However, most of the traditional segmentation methods are unsupervised, and they can extract objects based on observable or expressed features using prior knowledge. To make use of the advantages of both DNNs and traditional segmentation methods, in the cerebrovascular segmentation field, we combine traditional machine learning method with DNN models to achieve unsupervised DNN training scheme.

According to our experimental results, both HMRF + SegNet2D and HMRF + U-Net3D have good performances in cerebrovascular segmentation, and which are better than that of HMRF although they are trained with the results of HMRF. The accuracy and the specificity are both high, the accuracy of all three methods is above 0.99, and the specificity is above 0.999. But the sensitivity is quite different, sensitivity of HMRF method is just 0.5073, while that of the other two methods can reach a value above 0.76, which shows that the performance of HMRF with DNN method is much better than that of HMRF method. Though the accuracy is high, but the sensitivity is low. Low sensitivity and high accuracy is due to the imbalance of the negative and the positive samples. The proportion of blood vessels in human brain is small, so most samples are negative and a few are positive, which led to a large amount of negative samples and a small amount of positive samples. When calculating the accuracy, we used both true positive and true negative results as numerator, and all positive and negative samples as denominator, so the numerator is close to the denominator. But when calculating the sensitivity, only true positive is used as numerator, while the denominator is the sum of the true positive and false negative results. As the number of the negative samples is much larger than that of the positive samples, the false negative results is large due to the large base, that makes the sensitivity low.

The statistical results of the healthy people and stroke patients are similar, although the blood vessels are often smudged in stroke patients. The DSC is also similar and it is noticed that the DSC of stroke patients are even a little higher than that of the healthy people. As shown in Figures 6, 7, the number of vessels from the stroke patient is less than that of the healthy person, and the complexity of vascular distribution is low. We think that is why the stroke DSC value is similar to that of the healthy people, due to the fact that the details of stroke patients are not as much as those of the healthy people, which led to a better DSC value.

In fact, many small blood vessels are segmented by HMRF + SegNet2D and HMRF + U-Net3D, but not by HMRF. This can be explained from the view of feature extraction. In TOF-MRA images, different blood vessels share many similar features such as shapes, while their differences mainly are intensity contrast and vessel thickness. Since blood vessels segmented by HMRF are mainly large and high contrast vessels, DNN models mainly learn the features of large and high contrast vessels, while max-pooling layers in DNN provide a learning ability based on the different resolution features of blood vessels. Thus, DNN models trained by HMRF segmented blood vessels have stronger ability to recognize smaller blood vessels than HMRF method. Moreover, to improve the robustness of the proposed method for different kind of TOF-MRA images, we mixed 1.5T GE and 3.0T SIEMENS, healthy and ischemia stroke TOF-MRA images in training dataset.

Because of the limitation to obtain the manual annotations from public TOF-MRA dataset, it is difficult to directly compare our method to other DNN-based cerebrovascular segmentation methods, such as Y-Net (Chen et al., 2017) and CNN method proposed by Phellan et al. (2017). However, in terms of Dice numbers, our unsupervised method shows the great potential to perform automatic cerebrovascular segmentation.

In the future, we will investigate post-processing methods to boost the performance of the proposed method. Moreover, we will focus on the accurate segmentation of Willis circle and stenosis part of brain blood vessels since it can provide a fast and efficient stenosis detection method.

## Conclusion

We proposed a new unsupervised cerebrovascular segmentation framework based on HMRF model and DNN techniques in brain TOF-MRA images. The DNN model was trained by the label data obtained from HMRF model rather than manual annotations, which cost effective in terms of manual efforts. This cerebrovascular segmentation framework achieved a state-of-art performance evaluated on both 2D and 3D TOF-MRA images.

## Author Contributions

SF, YB, HC, and TT designed the model and method. QY provided the data and analyzed the results. SF and YB wrote the manuscript. YK and TT reviewed and edited the manuscript. All authors read and approved the manuscript.

## Conflict of Interest

SF, YB, and YK were employed by the Neusoft Institute of Intelligent Healthcare Technology, Co. Ltd.

The remaining authors declare that the research was conducted in the absence of any commercial or financial relationships that could be construed as a potential conflict of interest.
